# Reverse Solute Diffusion Enhances Sludge Dewatering in Dead-End Forward Osmosis

**DOI:** 10.3390/membranes14090196

**Published:** 2024-09-18

**Authors:** Da-Qi Cao, Shi-Cheng Lei, Hui Liu, Yan Jin, Yun-Feng Wu, Yuehua Cui, Rongling Wu

**Affiliations:** 1 Sino-Dutch R&D Centre for Future Wastewater Treatment Technologies/Key Laboratory of Urban Stormwater System and Water Environment, Beijing University of Civil Engineering and Architecture, Beijing 100044, China; 2 Department of Statistics and Probability, Michigan State University, East Lansing, MI 48824, USA; 3 Yau Mathematical Sciences Center, Tsinghua University, Beijing 100084, China; 4 Beijing Institute of Mathematical Sciences and Applications, Beijing 101408, China

**Keywords:** dead-end forward osmosis, sludge deep dewatering, reverse solute diffusion, extracellular polymer substances, osmosis resistance

## Abstract

Wastewater treatment plants produce high quantities of excess sludge. However, traditional sludge dewatering technology has high energy consumption and occupies a large area. Dead-end forward osmosis (DEFO) is an efficient and energy-saving deep dewatering technology for sludge. In this study, the reverse osmosis of salt ions in the draw solution was used to change the sludge cake structure and further reduce its moisture content in cake by releasing the bound water in cell. Three salts, NaCl, KCl, and CaCl_2_, were added to the excess sludge feed solution to explore the roles of the reverse osmosis of draw solutes in DEFO. When the added quantities of NaCl and CaCl_2_ were 15 and 10 mM, respectively, the moisture content of the sludge after dewatering decreased from 98.1% to 79.7% and 67.3%, respectively. However, KCl did not improve the sludge dewatering performance because of the “high K and low Na” phenomenon in biological cells. The water flux increased significantly for the binary draw solute involving NaCl and CaCl_2_ compared to the single draw solute. The extracellular polymer substances in the sludge changed the structure of the filter cake to improve the formation of water channels and decrease osmosis resistance, resulting in an increase in sludge dewatering efficiency. These findings provide support for improving the sludge dewatering performance of DEFO.

## 1. Introduction

The excess sludge generated in wastewater treatment plants (WWTPs) with high moisture content is difficult to treat and dispose of. The reduction and resource utilization of excess sludge are key components of sludge management technology and are important issues pertaining to the carbon neutrality of WWTP systems [1,2,3,4]. Recycling can be achieved through energy and material recovery. For example, biomethane is produced from anaerobic digestion sludge, and extracellular polymeric substances (EPSs) extracted from the sludge are used as metal adsorbents [5,6,7]. Efficient sludge deep dewatering reduces the burden of subsequent treatment and disposal.

With the gradual increase in domestic environmental awareness and stricter management laws and regulations, the requirements for the treatment and disposal of excess sludge have become stricter. Dewatering excess sludge is one of the most difficult and expensive processes in wastewater treatment [8,9]. Excess sludge has high moisture content even after concentration or digestion and needs to be further dehydrated to reduce the sludge volume. Sludge dewatering can reduce the moisture content of sludge to 65–85%. Traditional methods, such as mechanical dehydration, are usually accompanied by high energy consumption and a high carbon footprint [10,11]. Enhancement technologies such as chemical conditioning, heat treatment, and electric/magnetic field treatment are widely used to achieve sludge deep dewatering. Furthermore, high-pressure filtration and electromechanical dewatering have been used for sludge deep dewatering [12,13].

Traditional mechanical dehydration has disadvantages such as high energy consumption, high moisture content of the filter cake, a high carbon footprint, and poor effluent quality [14,15]. In most cases, the waste liquid produced after sludge treatment and disposal has poor water quality and it must be redirected to flow into the pipeline of the sewage treatment plant for secondary treatment or separate treatment [14,16]. These aspects increase the operation and management costs of traditional sludge dewatering methods [14,17,18,19,20]. Membrane separation is a cutting-edge technology used for sludge dewatering; however, heavy membrane fouling limits its widespread use. The dead-end forward osmosis (DEFO) process is considered a promising technology for sludge deep dewatering owing to its characteristics such as low energy consumption, high efficiency, and light membrane fouling [1,21].

Reverse solute diffusion (RSD) is regarded as the bottleneck in the development of the forward osmosis (FO) process. RSD is a unique phenomenon that cannot be avoided in the FO process, and it significantly affects the characteristics of the feed solution. Although it is one of the causes of membrane fouling [22,23], it also causes a decrease in the driving force and a loss of draw solutes [24,25]. The RSD in the FO process can be reduced by the addition of reagents, pressure, electrolysis, ultrasonic penetration, and developing new FO membranes and adopting new draw solutions [24,26,27,28,29].

The RSD changes the properties of the feed solution. Studies have shown that the reverse diffusion of Na^+^ leads to a reduction in electrostatic repulsion between protein and polysaccharide molecules, which transforms the gel network into a filter cake layer and exacerbates membrane fouling [30,31]. Monovalent cations affect the structure of the cake layer by influencing the molecular chain structure of the substances [30,32]. By promoting the complexation and crosslinking of EPS, bivalent cations can affect the formation of the filter cake layer and aggravate membrane fouling. Furthermore, EPSs have stronger binding selectivity for Ca^2+^ than for Mg^2+^ and are more likely to form a stable gel network with Ca^2+^ [33,34,35]. In addition, metal ions can change the structure of EPS. Compared to polyvalent cations, monovalent cations can reduce the density of EPSs and easily affect sludge dewatering performance [36]. Conversely, RSD promotes substance recovery during FO and achieves an effective concentration and recovery of alginate substances through the reverse diffusion of Ca^2+^ in the FO process [37,38]. Xu et al. found that RSD can increase the salinity of a microalgae solution [39]. The application of an electric-field-assisted system helped improve the electrochemical efficiency and increased the recovery efficiency of lipids in the microalgae solution. Kekre et al. combined electro-assisted technology with the FO process and promoted the RSD of Mg^2+^ in the draw solution by applying an electric field, which not only improved the water recovery rate but also promoted the recovery of guanite [40].

This study aims to clarify the role of draw solute reverse diffusion in sludge dewatering performance in DEFO. The filtration rate (*J*), water permeability coefficient (*A*), osmosis resistance (*R*_DEFO_), and filter cake moisture content (*R*) are used as evaluation parameters in DEFO. The effect of the reverse diffusion of Na^+^, K^+^, and Ca^2+^ in different concentrations of draw solutions on sludge dewatering performance was investigated. The sludge dewatering performances of various NaCl–CaCl_2_ binary draw solutions were compared to explore the synergistic effects of different draw solutes in sludge dewatering. The effects of EPSs on sludge dewatering were also investigated. To expand the source of the draw solution and the application scenario of the DEFO technology, simulated seawater, a simulated reverse osmosis concentrated solution of seawater, and Dalian seawater were used as draw solutions to explore their feasibility in DEFO sludge dewatering. Our study provides insights into improving the sludge DEFO dewatering rate through reverse osmosis using common salts, ultimately contributing to the optimization of DEFO technology for practical applications.

## 2. Materials and Methods

### 2.1. Materials

NaCl, KCl, CaCl_2_, MgCl_2_, NaHCO_3_, Na_2_SO_4_, and HNO_3_ (purity ≥ 99%) were purchased from Sinopharm Chemical Reagent Co. Ltd. (Shanghai, China). IR120Na-type cation-exchange resin (CER) was purchased from Rohm and Haas Co. (Philadelphia, PA, USA). Dialysis bags (molecular weight cutoff of 3500 Da) were purchased from Viskase Co., Inc. (Lombard, IL, USA). A cellulose triacetate embedded support FO (CTA-ES FO) membrane was purchased from Hydration Tech Innovations Co. (Albany, OR, USA).

Excess sludge was sourced from Beijing Gaobeidian and Beijing Dongba WWTPs, with the volatile suspended solid (VSS) and total solid contents of 57.5 ± 0.2% and 56.8 ± 0.3%, viscosities of 10.3 and 12.2 mPa·s, and pH of 8.0 and 7.1, respectively. The two types of sludge from Beijing Gaobeidian and Beijing Dongba WWTPs were denoted as G-S and D-S, respectively. After being subjected to standing at 25 °C for 24 h for the initial concentration, the sludge was stored in a refrigerator at 4 °C. The chemicals used in the experiments were of analytical grade, and all aqueous solutions were prepared using ultrapure water. EPSs were extracted from the excess sludge using the CER method as previously described [5]. Unless otherwise noted, the sludge used in this study is from Beijing Dongba WWTPs.

After removing the EPS from the sludge using the CER method, the remaining centrifugal sludge was filtered through a screen to extract the resin, which was further washed with the supernatant of the original sludge until the resin was cleaned. The cleaned sludge water was returned to the sludge. Subsequently, the excess sludge after the removal of EPS was centrifuged for 10 min at a rotational speed of 4000× *g*, and part of the supernatant was removed until the sludge concentration reached 18.75 g/L. The obtained sludge without EPS from Beijing Gaobeidian and Beijing Dongba WWTPs were denoted as G-S_EPS free_ and D-S_EPS free_, respectively.

### 2.2. Experimental Equipment and Operating Conditions

**Figure 1:**
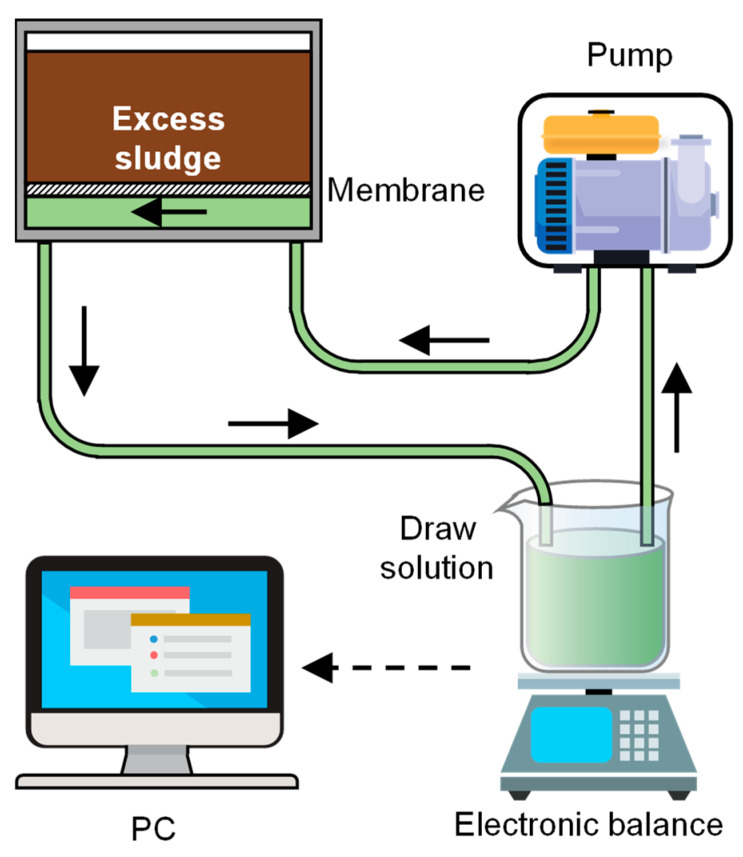
Schematic diagram of dead-end forward osmosis (DEFO) dewatering equipment.

The DEFO device was fabricated in the laboratory [1], and it consisted of an intelligent peristaltic pump, membrane assembly, electronic balance, beaker, pipeline, and computer, as shown in Figure 1. The DEFO adopts the operation mode of dead end on the feed solution side and cross-flow on the draw solution side. Its effective membrane area was 40.5 cm^2^. During the experiment, the active layer of the FO membrane was oriented toward the feed solution side to reduce membrane fouling during sludge dewatering.

To investigate the effect of the RSD of different draw solutions on DEFO dehydration, NaCl, KCl, and CaCl_2_ of different concentrations were added to the excess sludge, and after stirring for 20 min, a 1 M salt solution (NaCl, KCl, and CaCl_2_) was used as the draw solution, and the swept flow rate of the device was 3 cm/s. The synergistic effect of RSD of different draw solutions during the DEFO process was explored. The total concentration of the cations was maintained at 1 M, and binary draw solutions of NaCl and CaCl_2_ with different concentration ratios (1:0, 3:1, 1:1, 1:3, and 0:1) were prepared.

Simulated seawater (D1) and simulated concentrated seawater reverse osmosis (RO) solution (D2) were prepared as draw solutions to expand the DEFO application scenarios and draw solution sources [41]. Furthermore, practical seawater from Dalian (D3) was also used as a draw solution for sludge dewatering during the DEFO process.

### 2.3. Analytical Methods

#### 2.3.1. Measurement of Moisture and Metal Ions in Filter Cake

After the sludge was dehydrated for 10 h, the sludge filter cake was weighed in a dry crucible and baked in the oven at 105 °C for 12 h. The moisture content of the filter cake was calculated using the cake weights before and after dewatering. The cake was burned in a muffle furnace (SX2-4-10, Shanghai Yiheng Technology Co., Ltd., Shanghai, China) at 600 °C for 8 h to obtain ash. Then, 50% HNO_3_ solution was added to the ash at a solid/liquid ratio of 1:50, and the mixture was shaken for more than 4 h. After filtration, the ionic filtrate was extracted from ash via acid leaching. The accumulation of metal ions in the filter cakes was measured using an inductively coupled plasma emission spectrometer (ICAP 7000 Series, Thermo Fisher Scientific, Waltham, MA, USA).

#### 2.3.2. Physicochemical Properties of Sludge

The sludge filter cake was photographed using a mobile phone (Honor 30, Huawei Technologies Co., Ltd., Shenzhen, China). A rotating viscometer (HAAKE Viscotester 550, Thermo Fisher Scientific, Madison, WI, USA) with a DelsaNano C-type particle size/zeta potential analyzer (DelsaNano C type, Beckman Coulter Commercial, Pasadena, CA, USA) was used to measure the zeta potential of the sludge. The typical particle size distribution of colloidal EPS solutions was measured using dynamic light scattering with a laser particle size analyzer (Microtrac S3500, Microtrac Inc., York, PA, USA). The Lowry and phenol–sulfuric acid methods were used to measure proteins and polysaccharides in the EPS extracted from sludge by the CER method, respectively.

## 3. Results and Discussion

### 3.1. Dewatering Behaviors of Sludge with Single Salt

**Figure 2:**
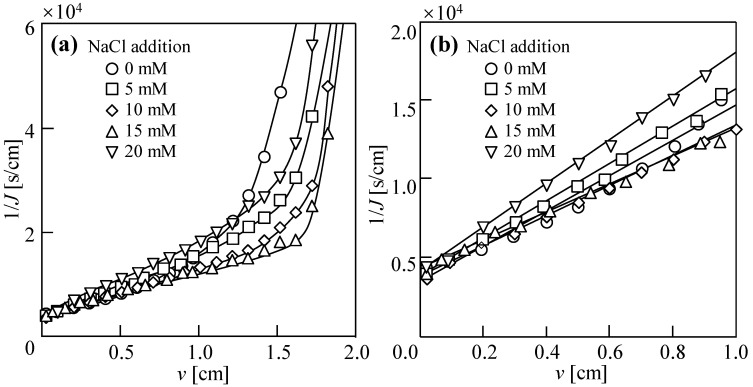
Dewatering behaviors of sludge with various NaCl concentrations in the dead-end forward osmosis (DEFO) process. (**a**) Filtration and compression stages. (**b**) Filtration stage. Herein, the concentration of feed solution sludge, *C*_FS_ = 18.75 g/L; cross-velocity on the draw side, *u* = 3.0 cm/s; concentration of draw solution, *C*_DS_ = 1.0 M NaCl.

Figure 2a shows the DEFO dewatering behavior (1/*J* vs. *v*) when a 1 M NaCl solution was used as the draw solution and the cross-velocity was 3 cm/s for 10 h. Here, 1/*J* shows the reciprocal of the water flux; *J* is the volume of water passing through the FO membrane area per unit time; *v* is the volume of water passing through the FO membrane per unit membrane area. Similar to traditional pressure filtration [42,43], DEFO sludge dewatering behaviors can be divided into the filtration stage and compression stage. With the addition of 0–15 mM of NaCl, 1/*J* exhibited a downward trend; that is, when the concentration of NaCl on the feed solution side increased within a certain range, the filtration rate gradually increased. When *v* was the same, with the addition of NaCl increasing within the range of 15–20 mM, 1/*J* showed an upward trend; that is, when the concentration of NaCl on the feed solution side increased beyond a certain range, the filtration rate decreased. When the added quantity of NaCl was 15 mM, the sludge dewatering stage had the lowest values of 1/*J*, namely, the water flux, *J*, was the highest. This indicates that appropriately increasing the quantity of the reverse diffusion of draw salts can improve the sludge dewatering performance. However, when the quantity of the reverse diffusion of draw salts was too high, the osmotic pressure difference on both sides of the membrane decreased quickly, resulting in poor sludge dewatering performance.

Figure 2b shows the dewatering behavior of the sludge during filtration. When 5–20 mM of NaCl was added, the filtration rate remained essentially unchanged and even a little lower compared to the scenario with no NaCl. In particular, the filtration rate for 20 mM NaCl was significantly higher than that for 15 mM NaCl, possibly because the RSD of NaCl led to changes in both factors such as osmotic pressure difference and sludge cake resistance. However, under the remaining NaCl concentration, the dewatering rate of the sludge was lower than that observed in the absence of NaCl. The difference in velocity between the different stages of sludge dehydration showed that the reverse diffusion of NaCl mainly played a role in the compression stage, which may have been due to the diffusion of NaCl changing the osmotic pressure difference inside and outside the sludge cells such that the bound water in the sludge was released.

**Figure 3:**
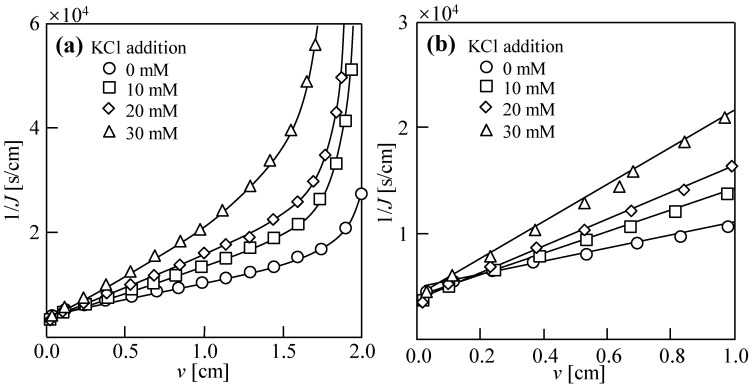
Dewatering behaviors of sludge with various KCl concentrations in dead-end forward osmosis (DEFO) process. (**a**) Filtration and compression stages. (**b**) Filtration stage. Herein, the concentration of feed solution sludge, *C*_FS_ = 18.75 g/L; cross-velocity on the draw side, *u* = 3.0 cm/s; concentration of draw solution, *C*_DS_ = 1.0 M KCl.

Figure 3 shows the dewatering behavior of sludge with various KCl concentrations when 1 M KCl solution was used as the draw solution. In contrast to the results of NaCl reverse diffusion, when *v* was the same, 1/*J* gradually increased with the increase in KCl in the range of 0–30 mM, and the filtration rate gradually decreased. The reverse diffusion of KCl did not enhance the dewatering performance of the DEFO sludge, probably because of two reasons. First, the higher quantity of reverse osmosis exhibited by KCl can be attributed to the small hydration radius of K^+^, resulting in a significant reduction in the osmotic pressure difference on both sides of the membrane and, consequently, a less effective dehydration process. Second, the prevalent “high K and low Na” phenomenon in biological cells necessitates the maintenance of ion concentration balance inside and outside the cell. The Na–K ion pump actively transports three Na^+^ ions out of the intracellular space while bringing two K^+^ into it [44,45]. When KCl reversely diffuses into the sludge, the increased KCl concentration among the sludge particles prompts the entry of K^+^ ions into the biological cells, perpetuating the “high K and low Na” phenomenon, rendering the release of intracellular bound water difficult.

**Figure 4:**
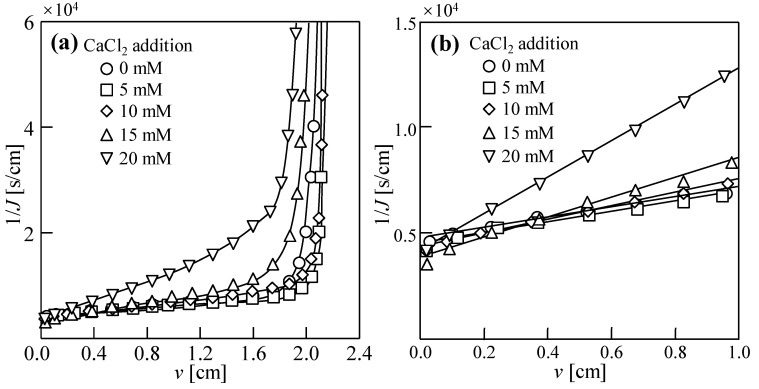
Dewatering behaviors of sludge with various CaCl_2_ concentrations in dead-end forward osmosis (DEFO) process. (**a**) Filtration and compression stages. (**b**) Filtration stage. Herein, the concentration of feed solution sludge, *C*_FS_ = 18.75 g/L; cross-velocity on the draw side, *u* = 3.0 cm/s; concentration of draw solution, *C*_DS_ = 1.0 M CaCl_2_.

Figure 4 shows the dewatering behavior of sludge with various CaCl_2_ concentrations when a 1 M CaCl_2_ solution was used as the draw solution. When the quantity of CaCl_2_ added was 5 mM, the 1/*J* during the thickening stage of sludge dehydration was the lowest, and the filtration rate was the highest. However, in contrast to the results of the NaCl reverse diffusion experiment, there was no clear relationship between the value of 1/*J* and the added quantity of CaCl_2_, probably because the radius of the hydrated ion of Ca^2+^ was larger than that of Na^+^ and blocked the pore of the membrane, leading to higher membrane fouling during the experiment.

**Figure 5:**
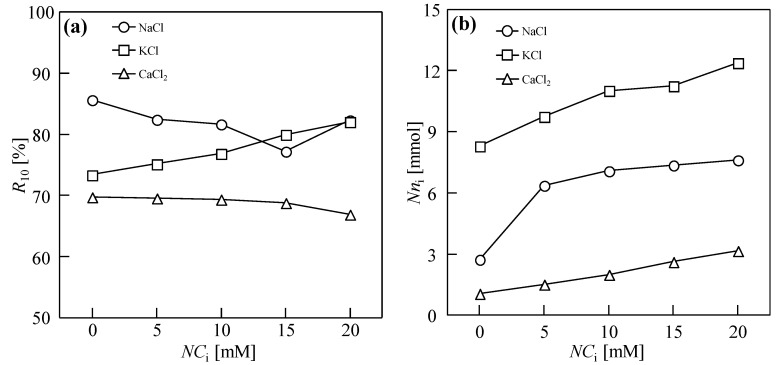
Sludge cake moisture content, *R*_10_ (**a**) and charge quantity of reverse cations diffusion, *Nn*_i_ (**b**) via 10 h dead-end forward osmosis for various types and concentrations of draw solutes. Herein, the abscissa is the total charge concentration, *NC*_i_, which is the product of the metal ion concentration (*C*_i_) and the ion charge number (*N*); *Nn*_i_ is the product of the quantity of reverse cation diffusion (*n*_i_) and the ion charge number (*N*); the concentration of sludge, *C*_FS_ = 18.75 g/L; cross-velocity on the draw side, *u* = 3.0 cm/s; concentration of draw solution, *C*_DS_ = 1.0 M.

Figure 5a shows the changes in moisture content in the filter cake under different NaCl, KCl, and CaCl_2_ addition quantities after sludge dewatering for 10 h. Here, $R_{10}=\frac{M_{1}-\;M_{2}}{M_{1}}\;\times \;100\%$, where *M*_1_ and *M*_2_ are the weights of the wet filter cake and dry cake before and after drying, respectively. When NaCl was used as the draw solution, the final moisture content of the filter cake initially decreased and then increased. The moisture content of the filter cake was the lowest (79.7%) when the added quantity was 15 mM. With an increase in the KCl, the *R*_10_ of the sludge cake increased gradually, proving that the reverse diffusion of KCl could not enhance the dewatering performance of the DEFO sludge. With increasing CaCl_2_, the moisture content first decreased and then increased. When the concentration was 10 mM, the moisture content of the cake was the lowest (67.3%).

The reverse osmosis quantity of cations in DEFO is shown in Figure 5b. The concentration of K^+^ ions in normal cells is much higher than that outside the cell, and the concentration of Na^+^ outside the cell is much higher than that inside the cell. First, because the radius of the hydrated K^+^ is the lowest, the quantity of the reverse osmosis of K^+^ in the FO process is the highest. To maintain a stable state of high K and low Na in the cell, the reverse diffusion of K^+^ quickly enters the cell through active transport and simultaneously weakens the concentration polarization of the FO membrane [44,45]. In contrast, the reverse osmosis of Na^+^ causes the extracellular Na concentration to become too high, causing cells to lose water. Similar to Na^+^, the reverse osmosis of Ca^2+^ changes the osmotic pressure of cells and causes them to lose water. On the other hand, Ca^2+^ can play a role in the compression of the double electric layer and bridge action [46,47] to further improve the sludge dewatering performance.

### 3.2. Water Permeability Coefficient and Osmosis Resistance in DEFO

As shown in Figure 2b, Figure 3b and Figure 4b, the relationships between 1/*J* and *v* show linearity in the filtration stage and can be evaluated by [1]

(1) $$\frac{1}{J}=\frac{2}{K}v+\frac{1}{J_{0}}$$

Here, *K* is the DEFO coefficient, which characterizes the difficulty of the DEFO dewatering of the sludge and is evaluated by

(2) $$K=\frac{2}{R_{\mathrm{DEFO}}}\cdot \frac{\pi }{\mu }$$

Here, *R*_DEFO_ is the osmosis resistance, which represents the water flow resistance caused by the FO membrane and filter cake on the FO membrane surface in DEFO; *π* is the osmotic pressure differential on both sides of the FO membrane, and *μ* is the viscosity of water. *J*_0_ is the initial value of *J* at time *t* = 0, evaluated by

(3) $$J_{0}=A\cdot \pi$$

where *A* is the water permeability coefficient, representing the permeability of the FO membrane.

**Figure 6:**
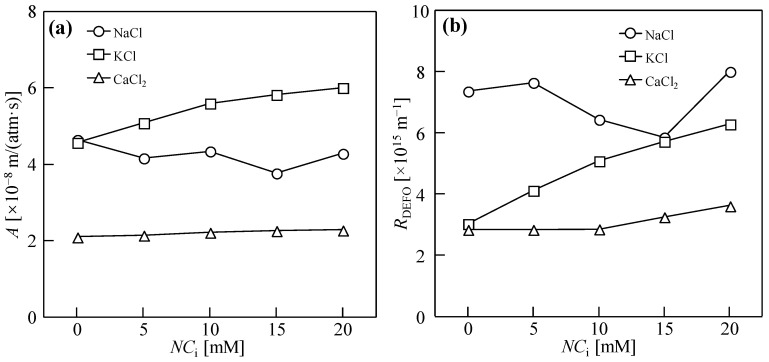
Water permeability coefficient, *A* (**a**) and osmosis resistance, *R*_DEFO_ (**b**) via 10 h of dead-end forward osmosis using various types and concentrations of draw solutes. Herein, the abscissa is the total charge concentration; *NC*_i_ is the product of the metal ion concentration (*C*_i_) and the ion charge number (*N*); the concentration of sludge, *C*_FS_ = 18.75 g/L; cross-velocity on the draw side, *u* = 3.0 cm/s; concentration of draw solution, *C*_DS_ = 1.0 M.

Figure 6 shows the water permeability coefficient, *A* and osmosis resistance, *R*_DEFO_. With an increase in NaCl addition, the *A* value maintained dynamic equilibrium overall, and *R*_DEFO_ showed a trend of first decreasing and then increasing. When the quantity of addition was 15 mM, the *R*_DEFO_ was the lowest at 5.9 × 10^15^ m^−1^. Therefore, an appropriate quantity of Na^+^ reverse diffusion can reduce the *R*_DEFO_ in the DEFO process and improve the sludge dewatering performance. With the addition of KCl, the *A* value continued to maintain dynamic equilibrium, but *R*_DEFO_ gradually increased. The increase in the quantity of KCl reverse diffusion directly led to the increase in the *R*_DEFO_ during the dehydration process, which may have been caused by the decrease in the driving force or the consolidation of the sludge cake. With the addition of CaCl_2_, the *A* value first increased and then decreased, whereas *R*_DEFO_ always increased. The results showed that the *A* value did not directly affect the final moisture content of the cake and that the increase in *R*_DEFO_ was caused by a decrease in the osmotic pressure difference between the two sides of the membrane, which jointly affected the dewatering performance of the cake.

### 3.3. Properties of Sludge Dewatering in DEFO with Binary Draw Solutes

**Figure 7:**
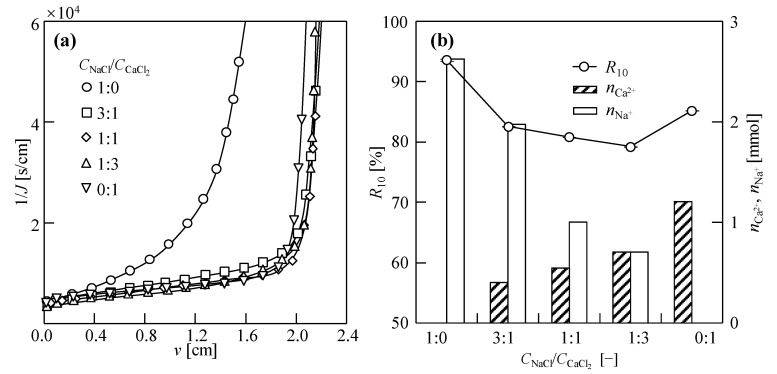
Dewatering behaviors of sludge (**a**) and osmosis resistance, *R*_DEFO_ and accumulation of cations in feed solutions (*n*_Ca_^2+^, *n*_Na_^+^) (**b**) in dead-end forward osmosis (DEFO) with various NaCl–CaCl_2_ binary draw solute ratios. Herein, the concentration of feed solution sludge, *C*_FS_ = 18.75 g/L; cross-velocity on the draw side, *u* = 3.0 cm/s; total concentration of draw solution, *C*_DS_ = 1.0 M.

Figure 7a shows the dewatering behavior (1/*J* vs. *v*) when a variety of NaCl–CaCl_2_ binary solutions (1 M) were used as draw solutions. When *v* was the same, 1/*J* first decreased and then increased with an increasing CaCl_2_ proportion in the binary draw solutions. Compared to the single draw solution (1 M NaCl), the filtration rate of the NaCl–CaCl_2_ draw solutions significantly improved during the DEFO process. In the compression stage, compared with the single draw solution (1 M CaCl_2_), the filtration rate was higher for the NaCl–CaCl_2_ draw solution. The filtration rate of the sludge DEFO dewatering process was the highest when the concentration ratio of NaCl to CaCl_2_ in the binary draw solution was 1:3. The results showed that when CaCl_2_ was added to the NaCl solution as a binary draw solution, the osmotic pressure of the solution increased and the sludge dewatering rate increased significantly. Furthermore, the addition of NaCl to CaCl_2_ to derive the binary draw solution alleviated the phenomenon of membrane pore blockage caused by the reverse diffusion of CaCl_2_. The reverse diffusion of NaCl and CaCl_2_ improved the dewatering rate of sludge in DEFO.

Figure 7b shows the cation accumulation and moisture content in the filter cake after 10 h of DEFO sludge dewatering. The extent of reverse diffusion of the draw solute is related to the concentration of ions in the draw solution. The moisture content in the case of the NaCl–CaCl_2_ draw solution was generally lower than that in the case of the single draw solution, and the moisture content decreased with an increase in the CaCl_2_ concentration ratio in the binary draw solution. When the concentration ratio of NaCl to CaCl_2_ in the binary draw solution was 1:3, the cake moisture content was the lowest (58.5%) and the sludge reduction effect increased by 67.1% compared with the NaCl draw solution and by 22.0% compared with the CaCl_2_ draw solution. The results show that the NaCl–CaCl_2_ draw solution can enhance the DEFO sludge dewatering efficiency, reduce the final moisture content of the filter cake, and improve the sludge dewatering performance. This enhancement effect is not only dependent on the increase in the osmotic pressure of the draw solution, but is also related to the synergistic effect of NaCl and CaCl_2_ on sludge flocs.

On the other hand, combined with the cost of the draw solutes (for example, the market prices of NaCl and CaCl_2_), the use of a binary mixed salt solution as the draw solution during sludge DEFO dewatering not only improved the DEFO dewatering performance but also reduced the cost of sludge treatment.

### 3.4. Effect of EPSs on Dewatering Behaviors of Sludge

**Figure 8:**
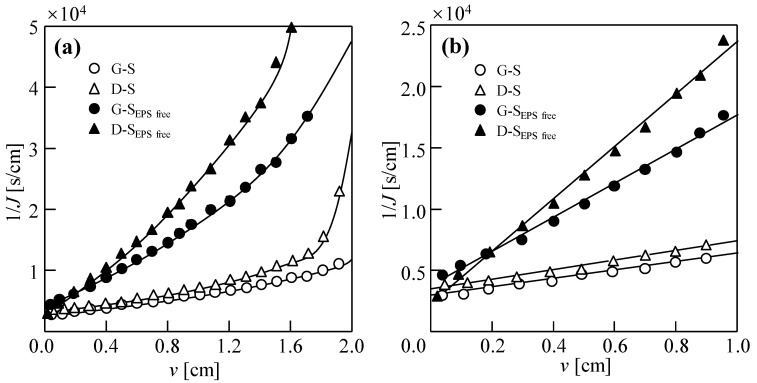
Dead-end forward osmosis dewatering behaviors of initial sludge and sludge without extracellular polymer substances (EPSs). (**a**) Filtration and compression stage; (**b**) filtration stage. Herein, the concentration of sludge, *C*_FS_ = 18.75 g/L; cross-velocity on the draw side, *u* = 3.0 cm/s; concentration of draw solution, *C*_DS_ = 2.0 M NaCl. G-S: sludge from Beijing Gaobeidian WWTPs; D-S: sludge from Beijing Dongba WWTPs; G-S_EPS free_: sludge obtained after removing the EPS from the sludge of Beijing Gaobeidian WWTPs using the CER method; D-S_EPS free_: sludge obtained after removing the EPS from the sludge of Beijing Dongba WWTPs using the CER method.

The EPSs in the excess sludge of the two WWTPs was removed, whereas the sludge concentration was maintained at 18.75 g/L for both G-S_EPS free_ and D-S_EPS free_. The dewatering behaviors of four types of sludge in DEFO are shown in Figure 8. After removing the EPS from the same type of sludge, when *v* was the same, 1/*J* increased and the filtration rate decreased. This indicates that an EPS in the sludge improves the dewatering performance by promoting sludge flocculation; therefore, the dewatering efficiency of the sludge decreases after removing the EPS.

**Figure 9:**
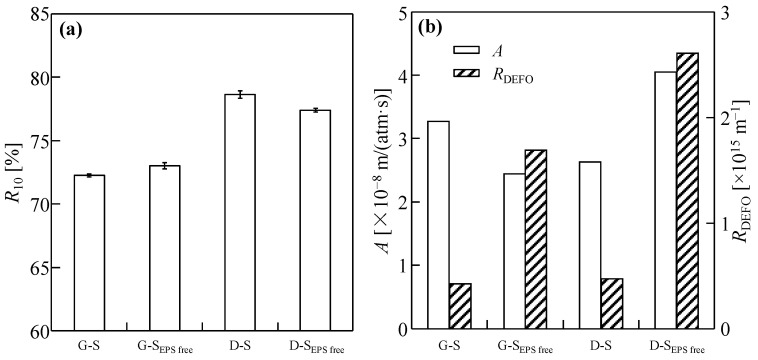
Sludge cake moisture content, *R*_10_ (**a**) and water permeability coefficient, *A* and osmosis resistance, *R*_DEFO_ (**b**) in the dead-end forward osmosis (DEFO) of the initial sludge and sludge without extracellular polymer substances (EPSs), calculated using data of Figure 8.

The moisture contents of the filter cakes after 10 h of dewatering of the four sludge types are shown in Figure 9a. Compared with the same kind of sludge, the cake moisture content of G-S_EPS free_ was slightly higher than that of the original sludge, while that of D-S_EPS free_ was slightly lower than that of the original sludge because sludge is an extremely complex substance, and many factors affect the moisture content of the filter cake in addition to the EPS content [48]. Overall, Gaobeidian sludge is easier to dewater than Dongba sludge, which may be due to the different wastewater treatment processes.

*R*_DEFO_ and *A,* assessed according to the DEFO velocity evaluation method in Section 3.2, are shown in Figure 9b. The *A* value of D-sludge gradually increased after the removal of the EPS, whereas that of G-sludge showed the opposite trend, probably due to varying contents of the main components in the EPS of the two types of sludge. After EPS removal, the *R*_DEFO_ of G-S_EPS free_ and D-S_EPS free_ increased significantly. Furthermore, the dewatering performances of the different sludge types also differed. The D-sludge was more affected by the EPS, and its *R*_DEFO_ increased more significantly. Further studies are required to analyze the changes in *R*_DEFO_ during the dewatering process of sludge DEFO.

Further analysis shows that the EPS content in the two types of sludge was similar, and the EPS content in the G-S was 123.2 mg/g VSS. The EPS content in the D-S was 122.3 mg/g VSS, and its main components were proteins and polysaccharides. The proportions of proteins and polysaccharides in the G-S EPS were 20.1 and 11.1%, respectively. For the D-S EPS, the proportions of proteins and polysaccharides were 26.8% and 9.0%, respectively. It was inferred that the proteins and polysaccharides in the EPS might affect *R*_DEFO_ during sludge dewatering. The composition of the EPS may be one of the reasons for the difference in *R*_DEFO_ during sludge dewatering. Because the EPS outside the sludge particles is in direct contact with RSD salt ions, it is likely to contribute to the main *R*_DEFO_ in the sludge dewatering process.

**Figure 10:**
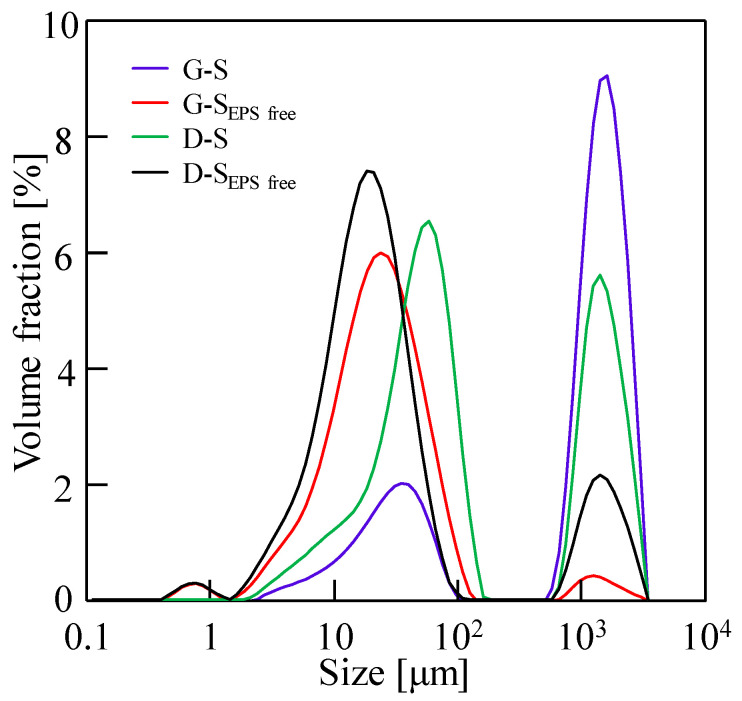
Typical particle size distributions of the initial sludge and the sludge without the EPS.

The physical and chemical properties of the sludge after EPS removal were further analyzed, and the pH, zeta potential, average particle size, and viscosity of the sludge are shown in Table 1. After removing the EPS, the pH of the sludge increased because EPSs contain a high number of functional groups, such as hydroxyl (-OH) and carboxylic (-COOH) groups, which produce H^+^ when ionized in water [5]. After the removal of the EPS, the absolute value of the zeta potential of the sludge increased, indicating that the electronegativity between sludge particles was enhanced and the flocculation ability of the sludge was weakened [49]. Figure 10 shows the typical size distributions of the four sludge types, and the average particle sizes are also shown in Table 1, indicating that the particle sizes in the sludge after the removal of the EPS decreased. Furthermore, the viscosities of the sludge without the EPS increased. Therefore, there were high zeta potential absolute values, small particle sizes, and high viscosity for the sludge without EPS, thereby increasing the osmosis resistance of the filter cake to water flow in the DEFO dehydration process.

**Figure 11:**
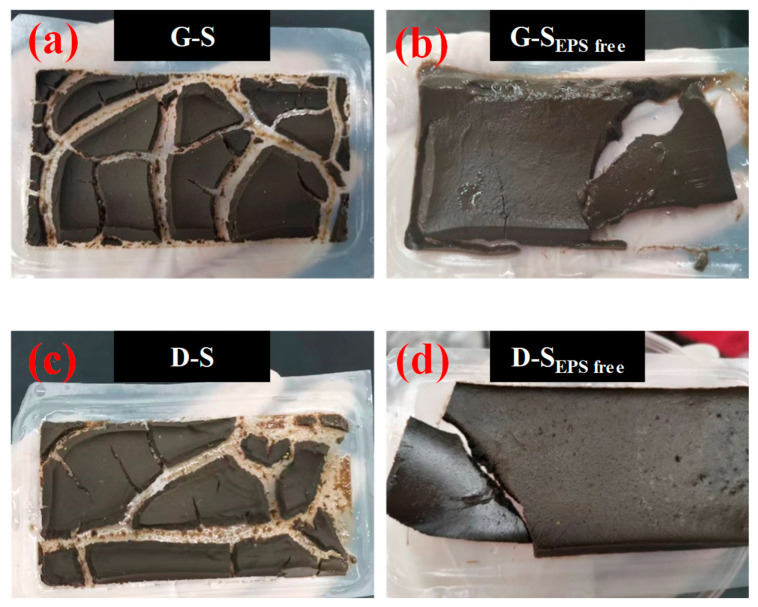
Typical pictures of filter cakes formed in dead-end forward osmosis dewatering for initial sludge and sludge without extracellular polymer substances (EPSs). (**a**) G-S; (**b**) D-S; (**c**) G-S_EPS free_; (**d**) D-S_EPS free_.

A previous study analyzed the morphology of filter cake and found that it presents a “dense top and loose bottom” structure during the progress of DEFO [1]. The water channel near the membrane surface helped water molecules flow from the sludge solution to the draw solution. However, a reduction in the sludge particle size may have led to the formation of a dense cake layer, which greatly increased the filtration *R*_DEFO_ and deteriorated the dewatering performance of the excess sludge. Figure 11 shows the morphologies of the filter cakes after the four types of sludge were dehydrated. Regardless of whether G-S_EPS free_ or D-S_EPS free_ was used, the sludge after the removal of EPS formed a continuous, uncracked filter cake, as shown in Figure 11b,d. No water channels between the filter cake layers were found, and the filter cake near the FO membrane surface was very dense. This proved that the presence of EPS in the sludge played an important role in the formation of water channels in the cake layers.

### 3.5. Mechanisms of DEFO Sludge Dewatering Enhancement

**Figure 12:**
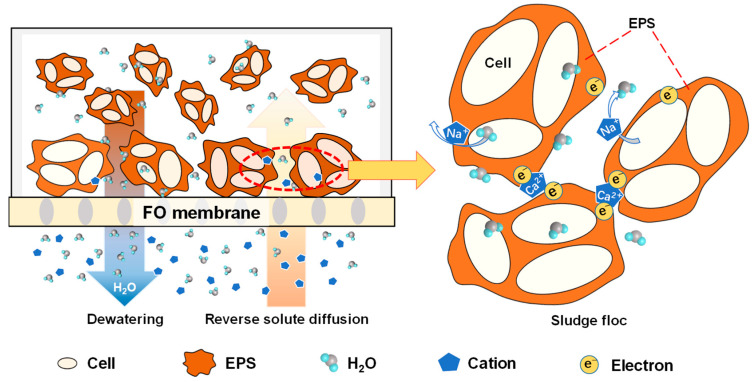
Schematic diagram showing mechanisms through which reverse solute diffusion improves the performance of sludge deep dewatering in the dead-end forward osmosis (DEFO) process. Here, EPS is extracellular polymer substance.

Figure 12 shows the schematic diagram showing mechanisms through which reverse solute diffusion improves the performance of sludge deep dewatering in the DEFO process. Using the osmotic pressure difference on both sides of the FO membrane as the driving force, water molecules in the sludge feed solution are drawn from the feed side to the draw side, resulting in sludge dewatering. At the same time, the reverse diffused solutes from draw solution interact with the sludge particles to promote sludge dewatering. For example, as shown in Figure 12, the draw solutes such as NaCl and CaCl_2_ diffused into the sludge simultaneously and cooperatively promoted sludge dewatering. The diffusion of draw solutes changes the osmotic pressure difference inside and outside the sludge cells, resulting in the release of bound water in cells. The diffusion of bivalent cations such as Ca^2+^ promotes the flocculation of sludge particles through the compression of the double electric layer and the bridging action [46,47], resulting in the low osmosis resistance of the filter cake formed by sludge. In addition, the presence of EPSs in sludge also alters the interaction of reverse permeable ions with the sludge flocs, mainly by changing the sludge floc charge, the size of the sludge floc particles, the sludge viscosity, and the structure of the sludge filter cake.

### 3.6. Effectivity of Practical Draw Solutions

**Figure 13:**
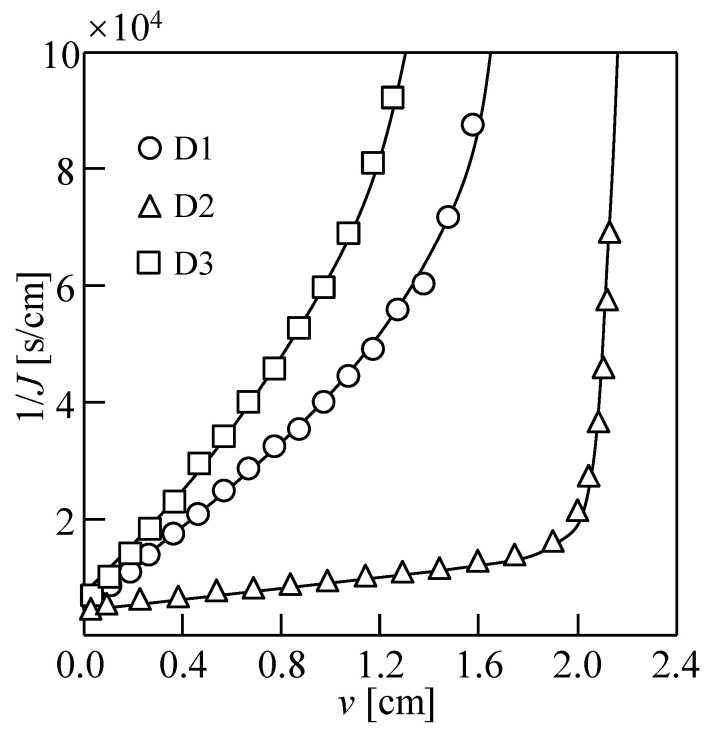
Dewatering behaviors of sludge with different types of seawater draw solutions as draw solutions. Here, D1: simulated seawater, D2: simulated concentrated seawater reverse osmosis solution, D3: practical seawater from Dalian; the concentration of sludge, *C*_FS_ = 18.75 g/L; cross-velocity on the draw side, *u* = 3.0 cm/s.

The dewatering behaviors (1/*J* vs. *v*) during 20 h of DEFO with different types of practical draw solutions are shown in Figure 13. D1, D2, and D3 were used to explore the feasibility of dewatering DEFO sludge under different application scenarios. When *v* was the same, the filtration rate, *J* for practical Dalian seawater was lower than that for simulated seawater. However, as shown in Table 2, the moisture contents of the filter cake after 20 h of DEFO dewatering using simulated seawater (D1) and practical Dalian seawater (D3) were 90.3% and 88.4%, respectively, indicating that the practical seawater has better sludge dewatering performance, probably because a wider variety of draw solutes in practical seawater interact with the sludge feed solution after RSD. Clearly, the simulated seawater RO-concentrated solution (D2) exhibited good dewatering performance with a maximum water flux and the filter cake moisture content of 65.7% because of the high concentration of draw solution.

Through the proper source selection of a draw solution, the cost of the sludge deep dewatering process via the proposed DEFO with the use of RSD will be further reduced. For example, DEFO sludge dewatering technology in coastal areas can be combined with the reverse osmosis process of seawater desalination. The concentrated reverse osmosis solution after seawater desalination was selected as the draw solution to dilute the high-concentration brine generated during the desalination process, and the sludge was subjected to deep dewatering. This reduces the impact of the direct discharge of this high-concentration brine on the ecological environment. In addition to the seawater draw solution, high-concentration fertilizers can be used as a source of DEFO draw solution [50], which can be directly used for agricultural irrigation after sludge dehydration and draw solution dilution.

## 4. Conclusions

In this study, the influence of the reverse diffusion of different solutes (NaCl, KCl, and CaCl_2_) on the dewatering of DEFO sludge was explored. The reverse osmosis of NaCl and CaCl_2_ improved the sludge DEFO dewatering rate. At optimal quantities of 15 and 10 mM of NaCl and CaCl_2_, respectively, the moisture content of the sludge decreased from 98.1% to 79.7% and 67.3%, respectively. The addition of KCl did not improve the dewatering performance of the sludge because of the “high K and low Na” phenomenon observed in biological cells. The NaCl–CaCl_2_ binary draw solution at the concentration ratio of NaCl: CaCl_2_ = 1:3 exhibited the lowest moisture content (58.5%) because of the synergistic effect between these components. The presence of EPS in excess sludge changes its particle size distribution, charge properties, sludge viscosity, and filter cake structure, resulting in the lighter osmosis resistance and better sludge dewatering efficiency. The moisture content of the filter cake was 65.7% when concentrated seawater was used as the draw solution. In the future, various practical draw solutions and novel FO membranes and their stability will be investigated for use in DEFO sludge dewatering.

## Figures and Tables

**Table 1:** Properties of four types of sludge.

Sludge Sources	pH	Zeta Potential [eV]	Average Particle Size [µm]	Viscosity [mPa·s]
G-S	8.0	−19.7	1194.4	10.3
G-S_EPS free_	8.3	−26.2	73.5	11.6
D-S	7.1	−22.4	583.0	12.2
D-S_EPS free_	8.2	−24.3	236.0	15.6

**Table 2:** Composition of draw solutes and their dewatering properties in DEFO.

Draw Solution	NaCl [mg/L]	CaCl_2_ [mg/L]	MgCl_2_·6H_2_O [mg/L]	NaHCO_3_ [mg/L]	Na_2_SO_4_ [mg/L]	*R* [%]
D1	25,053	1215	11,946	192	3168	90.3
D2	35,790	1735	17,066	274	4526	65.7
D3	–	–	–	–	–	88.4

D1: simulated seawater; D2: simulated seawater reverse osmosis concentrated solution; D3: Dalian seawater; *R*: moisture content of filter cake after dewatering via DEFO; concentration of sludge, *C*_FS_ = 18.75 g/L; cross-velocity on the draw side, *u* = 3.0 cm/s; DEFO time for D1 and D3 is 20 h; DEFO time for D2 is 10 h.

## Data Availability

Data are contained within the article.
